# Comparative Study of the Fatty Acid and Phenolic Profiles of Tender and Mature Coconut for Coconut Milk Production

**DOI:** 10.3390/foods14234023

**Published:** 2025-11-24

**Authors:** Rongqian Jiang, Danpeng Xue, Yanqing Chen, Xucong Lv, Li Ni, Zhibin Liu

**Affiliations:** 1 Food Nutrition and Health Research Center, School of Advanced Manufacturing, Fuzhou University, Jinjiang 362200, China; 2 Institute of Food Science & Technology, Fuzhou University, Fuzhou 350108, China

**Keywords:** coconut milk, fatty acid profile, mature coconut, phenolic profile, tender coconut

## Abstract

Coconut milk is a widely consumed plant-based milk alternative, valued for its creamy texture and functional properties. This study systematically evaluated the fatty acid and phenolic profiles of coconut meat and water at tender and mature stages to inform coconut milk production. Fatty acid analysis revealed that mature coconut meat (MCM) contained 299.7 g/kg total fatty acids, predominantly lauric acid (C12:0, 142.97 g/kg, 48%), myristic acid (C14:0, 57.39 g/kg, 19%), and palmitic acid (C16:0, 29.79 g/kg, 10%), whereas tender coconut meat (TCM) contained 90.87 g/kg total fatty acids. Tender coconut water (TCW) exhibited the highest total phenolic content (TPC) and DPPH radical scavenging activity. UHPLC-Q-Orbitrap-MS identified 1065 phytochemicals, including 96 phenolics, with multivariate analyses showing distinct profiles between tissues and developmental stages. Notably, tender samples contained higher levels of bioactive phenolics, such as catechin, epicatechin, and astilbin. Collectively, these results demonstrate that tissue type and developmental stage jointly shape the nutritional and functional attributes of coconuts. Mature coconut meat provides lipid-rich nutrition for coconut milk, while tender coconut water offers antioxidant-rich bioactivity. Integrating these raw materials can enhance the nutritional and functional properties of coconut milk, enhancing its role as a versatile plant-based alternative for functional food and beverages.

## 1. Introduction

In recent years, the global shift toward plant-based diets has accelerated, driven by growing concerns over environmental sustainability, animal welfare, and human health. Plant-based alternatives have become integral to the sustainable transformation of the global food system, primarily owing to their reduced greenhouse gas emissions, lower resource demands, and alignment with ethical and health-conscious consumer preferences [1]. Among these alternatives, plant-based milk substitutes have garnered substantial attention, particularly among individuals with lactose intolerance or dietary restrictions related to cholesterol and saturated fats [2]. Coconut milk, in particular, has emerged as a distinctive and versatile option, valued for its rich flavor, creamy mouthfeel, and unique nutritional composition. Its incorporation into beverages, particularly coffee-based products, has grown rapidly in markets such as China. Market analyses from Fortune Business Insights indicate substantial expansion in the global coconut milk market, with a projected compound annual growth rate (CAGR) of around 12% leading to a valuation of USD 7.2 billion by 2032, thus confirming its increasing importance in the realm of plant-based alternatives.

Coconut milk is a white, emulsion-based liquid extracted from the grated endosperm of the coconut fruit (coconut meat). It typically contains high fat (~33.5 g/100 g), high carbohydrates (~15.2 g/100 g), moderate sugars (~6.2 g/100 g), and low protein (~3.3 g/100 g) according to USDA data [3]. Notably, coconut milk is exceptionally rich in medium-chain fatty acids (MCFAs), particularly lauric acid (C12:0), which accounts for 45–50% of total fat. Other MCFAs, including myristic (C14:0), palmitic (C16:0), caprylic (C8:0), and capric (C10:0) acids [3], further contribute to its unique lipid composition.

Unlike long-chain fatty acids (LCFAs) predominant in dairy and most plant-based milks, MCFAs are directly absorbed into the portal circulation and rapidly metabolized to acetyl-CoA or ketone bodies [4,5]. This metabolic pathway bypasses the more complex digestion and transport mechanisms required for LCFAs [6], enabling faster energy release and potentially contributing to reduced fat accumulation and improved metabolic health. In contrast, cow’s milk and other plant-based milks (e.g., soy, almond, oat) primarily contain LCFAs such as palmitic (C16:0), stearic (C18:0), oleic (C18:1), linoleic (C18:2), and α-linolenic (C18:3) acids with lower total fat content (0.9–4.0 g/100 g). Thus, the MCFA-rich lipid profile distinguishes coconut milk in both metabolism and potential health functionality.

Beyond its lipid profile, coconut milk contains diverse bioactive phytochemicals, particularly phenolic compounds, which contribute to its antioxidant activity and potential health benefits [7]. These compounds include phenolic acids (e.g., gallic acid, caffeic acid, *p*-hydroxybenzoic acid, chlorogenic acid, ferulic acid, and syringic acid) and flavonoids such as catechin [8]. Previous studies, such as those by Arivalagan et al. [9] and Mahayothee et al. [10], have identified and quantified these compounds in coconut meat. Nevertheless, detailed characterization of these compounds remains limited, particularly regarding their variation across different geographic origins and maturity stages of the coconut fruit. Moreover, much of the existing literature relies on conventional analytical techniques with limited sensitivity and resolution. The advancement of high-resolution analytical technologies such as ultrahigh-performance liquid chromatography coupled with quadrupole-Orbitrap high-resolution mass spectrometry (UHPLC-Q-Orbitrap-MS) now allows for more comprehensive and precise characterization of complex phenolic profiles in plant-derived foods [11]. These techniques offer significant advantages over traditional LC-MS methods in terms of sensitivity, resolution, and structural elucidation capabilities, thereby providing new opportunities for in-depth exploration of coconut milk’s phytochemical diversity.

Another key factor influencing coconut milk composition is the maturity of the raw material. Commercial production typically uses mature coconut meat (~12 months after flowering), while coconut water, especially from tender coconuts (~8 months), is often discarded despite evidence of its high phenolic content and antioxidant activity [12]. Understanding how fruit maturity and tissue type (meat vs. water) jointly affect fatty acid and phenolic profiles could inform improved resource utilization and product formulation.

Therefore, the current study aims to comprehensively analyze the fatty acid and phenolic profiles of coconut meat and coconut water from the tender coconuts and the mature coconuts. Fatty acid composition was qualitatively and quantitatively examined using gas chromatography–mass spectrometry (GC–MS), while phenolic compounds were characterized using UHPLC–Q-Orbitrap–MS. By integrating these analyses, this work elucidates the developmental chemistry of coconuts and evaluates the potential of coconut water as a functional ingredient in coconut milk production. The findings are expected to enhance understanding of coconut milk’s compositional diversity and guide the development of nutritionally optimized, plant-based functional beverages.

## 2. Materials and Methods

### 2.1. Samples and Reagents

Coconut fruits (*Cocos nucifera* L., dwarf variety) were collected at two maturity stages: tender coconuts harvested 8 months after flowering (n = 10) and mature coconuts harvested 12 months after flowering (n = 15). After removing the shells, the coconut meat was separated, chopped, and homogenized with distilled water at a ratio of 1:4 (*w*/*v*) using a colloid mill. The homogenate was filtered to obtain a white, creamy slurry. The two types of coconut meat were designated as TCM (tender coconut meat) and MCM (mature coconut meat). Corresponding coconut water samples were collected and labeled as TCW (tender coconut water) and MCW (mature coconut water). All samples were freeze-dried and stored at −20 °C until analysis.

Analytical-grade reagents and solvents, including chloroform, n-hexane, methanol, gallic acid, and anhydrous calcium carbonate, were purchased from Sinopharm Chemical Reagent Co., Ltd. (Shanghai, China). Sodium hydroxide, sodium chloride, boron trifluoride etherate, sodium bicarbonate, and phosphoric acid were purchased from Xilong Chemical Co., Ltd. (Shenyan, China). Folin–Ciocalteu reagent and 1,1-diphenyl-2-trinitrohydrazine (DPPH) were purchased from MacLean Biochemical Technology Co., Ltd. (Shanghai, China). A fatty acid methyl ester mixed standard was purchased from Tanmo Quality Inspection-Standard Material Center (Beijing, China). Formic acid (LC-MS grade) was obtained from CNW Technologies GmbH (Düsseldorf, Germany); LC-MS grade acetonitrile and methanol were purchased from Fisher Chemical (Pittsburgh, PA, USA). Ultrapure water was obtained from a Milli-Q purification system (Millipore, MA, USA).

### 2.2. Lipid Extraction, Methyl Esterification, and GC–MS Analysis

Lipid extraction followed the method established by Mahayothee et al. [10], with slight modifications. Briefly, 60 mg of freeze-dried sample was placed in a 10 mL centrifuge tube and extracted three times with 6 mL chloroform–methanol (2:1, *v*/*v*). During each extraction, the mixture was vortexed for 3 h, followed by centrifugation (12,000 rpm, 15 min). The combined supernatants were evaporated under nitrogen to remove residual solvents, and the lipid extracts were stored at −20 °C until analysis.

Fatty acid methyl esters were prepared following the method described in EN ISO 12966-2:2017 Part 2: Preparation of methyl esters of fatty acids [13]. The dried lipid extract was reacted with 10 mL of 0.5 mol/L sodium hydroxide–methanol solution in a shaking water bath at 200 rpm and 70 °C for 20 min. After cooling, 10 mL of boron trifluoride–methanol solution (prepared at a 1:3 ratio of anhydrous methanol to boron trifluoride etherate) was added, and the mixture was shaken for another 20 min. After cooling the reaction mixture, it was extracted with 2 mL of n-hexane and vortexed for 2 min. We then added a saturated sodium chloride solution, collected the resulting upper hexane phase, and filtered (0.45 μm PTFE filter) it before GC-MS analysis.

GC–MS conditions were as follows: injector temperature, 300 °C; detector temperature, 280 °C; injection mode, split (split ratio 1:50); injection volume, 1 μL. The oven temperature program: 80 °C (hold 2 min), ramped to 250 °C at 5 °C/min (hold 5 min), then ramped to 300 °C at 10 °C/min (hold 9 min). The ion source temperature was 280 °C, interface temperature 300 °C, with a solvent delay of 2 min. A total of nine fatty acids were identified in all samples, and quantification was performed using external standards. Calibration curves for the nine fatty acids are provided in Supplementary Table S1.

The nutritional and health-related lipid indices, including the hypocholesterolemic/hypercholesterolemic ratio (H/H) and the indices of atherogenicity (IA) and thrombogenicity (IT), were calculated based on the fatty acid composition of each sample.

The H/H ratio was calculated according to Santos-Silva et al. using the following equation [14]:

$$H/H=\frac{\Sigma MUFA+\Sigma PUFA}{C12:0+C14:0+C16:0}$$

The indices of IA and IT were calculated according to Ulbricht and Southgate using the following equations [15]:

$$IA=\frac{C12:0+4\times C14:0+C16:0}{\Sigma MUFA+{\Sigma PUFA}_{n-6}+{\Sigma PUFA}_{n-3}}$$

$$IT=\frac{C14:0+C16:0+C18:0}{0.5\times \Sigma MUFA+0.5\times {\Sigma PUFA}_{n-6}+3\times {\Sigma PUFA}_{n-3}+\frac{{\Sigma PUFA}_{n-3}}{{\Sigma PUFA}_{n-6}}}$$

All fatty acids were expressed as a percentage of total identified fatty acids prior to calculation.

### 2.3. Determination of Total Phenolic Content (TPC) and DPPH Radical Scavenging Activity

TPC was examined using the Folin–Ciocalteu method [11]. Briefly, 0.5 mL of extract was mixed with 0.25 mL Folin–Ciocalteu reagent, followed by the addition of 4 mL distilled water and 0.5 mL saturated Na_2_CO_3_ solution. The mixture was incubated in the dark for 1 h, after which its absorbance was measured at 725nm using a microplate reader. Gallic acid served as the standard, and the resulting values were calculated and expressed as gallic acid equivalents (GAE). Quantification was based on a calibration curve with the regression equation: Y = 0.009x + 0.0007 (R^2^ = 0.9998).

DPPH radical scavenging activity was assessed according to the method reported by Chen et al. [16]. A stock solution of DPPH (0.4 mmol/L) was prepared in methanol and diluted tenfold with deionized water before use. Equal volumes (10 mL) of sample extract and DPPH solution were mixed and incubated in the dark for 30 min at room temperature (25 °C). Absorbance was recorded at 517 nm. Gallic acid was used as the positive control, and results were expressed as GAE. The calibration curve equation was: Y = −0.0066x + 0.3557 (R^2^ = 0.9929).

### 2.4. LC-HRMS Analysis

Phenolic profiling was performed using UHPLC–Q-Orbitrap-MS [17]. Chromatographic separation was achieved with an ACQUITY UPLC HSS T3 column (100 mm × 2.1 mm, 1.8 μm; Waters, Milford, MA, USA). The mobile phase consisted of (A) 95% water + 5% acetonitrile with 0.1% formic acid, and (B) 47.5% acetonitrile + 47.5% isopropanol + 5% water with 0.1% formic acid. The injection volume was 3 μL, and the column temperature was maintained at 40 °C. The gradient program was as follows: 0 min, 100% A; 0–3 min, 80% A; 3–4.5 min, 65% A; 5–6.3 min, 100% B; 6.3–6.4 min, 100% B; 6.4–8 min, re-equilibration at 100% A.

Mass spectrometry was performed with electrospray ionization (ESI) in both positive and negative ion modes. The parameters were: scan range, 70–1050 *m*/*z*; sheath gas flow, 50 arb; auxiliary gas flow, 13 arb; capillary temperature, 325 °C; heater temperature, 425 °C; spray voltage, ±3500 V; S-lens voltage, 50 V. Collision energies were set at 20, 40, and 60 eV. The resolution was 60,000 (full MS) and 17,500 (MS^2^).

The raw data acquired from the UHPLC-Q-Orbitrap-MS was imported into the software Compound Discoverer (version 2.0, Thermo Fisher Scientific, Bremen, Germany) for peak extraction, alignment, and integration. This yielded a data matrix detailing information such as retention time, mass-to-charge ratio (*m*/*z*), and peak intensity. The software proceeded to search and identify feature peaks by comparing the MS and MS/MS spectral data against established metabolite databases, including HMDB, METLIN, and M/Zcloud, as well as an in-house metabolite database (Shanghai Majorbio Co., Ltd., Shanghai, China). The MS mass error was set to less than 10 ppm.

### 2.5. Statistical Analysis

All data are reported as mean ± standard deviation (SD). Duncan’s multiple range test was employed using SPSS software (Version 24.0.0.2, IBM, NY, USA) to determine statistical significance between groups, with a significance level set at *p* < 0.05. Furthermore, multivariate statistical analyses, including principal coordinate analysis (PCoA), hierarchical cluster analysis (HCA), and partial least squares-discriminant analysis (PLS-DA), were performed using R software (Version 4.5.1) to investigate variations in phenolic profiles based on maturity stage and tissue type.

## 3. Results and Discussion

### 3.1. Fatty Acid Profile Analysis

The fatty acid compositions of two freeze-dried coconut meat samples and two freeze-dried coconut water samples were analyzed by GC–MS following lipid extraction and methyl esterification. A total of nine fatty acids were identified across all samples: caproic acid (C6:0), caprylic acid (C8:0), capric acid (C10:0), lauric acid (C12:0), myristic acid (C14:0), palmitic acid (C16:0), stearic acid (C18:0), oleic acid (C18:1), and linoleic acid (C18:2). The representative total ion GC-MS chromatograms of the four types of samples were demonstrated in Supplementary Material Figure S1. Among the identified fatty acids, five (C6–C14) were classified as medium- and short-chain saturated fatty acids, while the remaining four (C16–C18) were long-chain fatty acids. All nine fatty acids were detected in coconut meat samples (MCM and TCM); however, caproic acid was absent in mature coconut water (MCW), and both caproic and caprylic acids were undetected in tender coconut water (TCW).

Quantitative analysis based on authentic standards revealed marked differences in total fatty acid content between tender and mature coconuts (Table 1). On a dry-weight basis, MCM contained 299.70 ± 16.98 g/kg of total fatty acids, more than three times higher than the 90.87 ± 16.86 g/kg observed in TCM. Despite these quantitative differences, the overall fatty acid profiles were broadly similar across maturity stages, with lauric, myristic, and palmitic acids being the predominant components. Lauric acid was the most abundant, with a concentration of 142.97 ± 4.99 g/kg in MCM, accounting for 48% of the total fatty acids, compared with 35.11 ± 5.49 g/kg (39%) in TCM. Thus, lauric acid levels in MCM were approximately 4.1 times higher than in TCM. Myristic acid ranked second, with concentrations of 57.39 ± 2.08 g/kg (19%) in MCM and 18.17 ± 0.61 g/kg (20%) in TCM, representing a 3.2-fold difference. Palmitic acid, the third most abundant fatty acid, was also significantly higher in MCM (29.79 ± 1.89 g/kg) than in TCM (12.53 ± 2.22 g/kg), a 2.4-fold increase. It has also been revealed by Guo et al. that multiple lipid biosynthesis-related genes, such as WRI3, WIN1, and SHN2, accumulated with the maturation of coconut meat [8]. By contrast, coconut water samples contained only trace levels of fatty acids, with concentrations of 3.6 ± 0.20 g/100 mL in MCW and 1.2 ± 0.20 g/100 mL in TCW, values negligible compared with those of coconut meat.

Given the nutritional relevance of medium-chain fatty acids (MCFAs) as rapidly metabolizable energy sources, their relative abundance was further examined. In MCM, MCFAs accounted for 59.51% of total fatty acids, compared with 45.49% in TCM. In coconut water, MCFA proportions were comparable between maturity stages, at ~50% in both MCW and TCW. These results suggest that coconut maturation strongly enhances the relative abundance of MCFAs in coconut meat, whereas the compositional profile of coconut water remains largely unaffected. This observation is consistent with previous studies. For example, Mahayothee et al. [10] reported significant increases in caproic, caprylic, capric, and lauric acids as coconuts matured from 180–190 days to 225 days after pollination in Thailand. Similarly, Kumar et al. [18] found that coconuts harvested at 11–12 months contained substantially higher proportions of capric, caprylic, and lauric acids than those harvested at 7–8 months, while the proportions of palmitic, oleic, and linoleic acids decreased. Together, these findings confirm that the compositional shift toward MCFA enrichment is a characteristic feature of coconut maturation.

The ratio between saturated fatty acids (SFAs) and unsaturated fatty acids (UFAs) also serves as an important indicator of lipid functionality. In the present study, SFAs dominated the fatty acid profile of all samples. Their relative proportions were 93.89% in MCM, 82.91% in TCM, 90.13% in MCW, and 86.19% in TCW. Therefore, SFAs are the major lipid constituents of both coconut milk and coconut water.

Additionally, the nutritional and health-related lipid indices derived from the fatty acid profiles showed distinct differences across coconut tissues and maturity stages. The hypocholesterolemic/hypercholesterolemic ratio (H/H) was highest in tender coconut meat and water (0.24 and 0.20, respectively), indicating relatively more favorable fatty acid compositions compared with mature samples. In contrast, MCM exhibited the lowest H/H value (0.08), reflecting its high proportion of hypercholesterolemic saturated fatty acids such as C14:0 and C16:0. The atherogenicity (IA) and thrombogenicity (IT) indices showed similar trends. MCM displayed markedly higher IA (20.81) and the lowest IT (433), while TCM had the lowest IA (7.75). TCW showed the highest IT (1650), suggesting that although TCW contains a comparatively healthier balance of unsaturated to saturated fatty acids, its specific fatty acid profile increases the thrombogenic potential relative to other tissues. Overall, these indices highlight that tender coconut tissues, particularly TCM, present a more favorable lipid-related nutritional profile, whereas mature coconut meat demonstrates less desirable characteristics due to its elevated levels of atherogenic and hypercholesterolemic fatty acids.

In summary, both coconut maturity and tissue type significantly influence fatty acid composition. As coconuts develop from tender to mature stages, the total lipid content increases markedly, accompanied by a relative enrichment of MCFAs, including lauric, caprylic, and capric acids, and a corresponding reduction in long-chain and unsaturated fatty acids. Consequently, the proportion of SFAs increases while that of UFAs declines. This developmental shift is likely associated with the upregulation of specific genes involved in medium-chain fatty acid biosynthesis during coconut maturation.

### 3.2. Total Phenolic Content and DPPH Scavenging Activities Analysis

**Figure 1:**
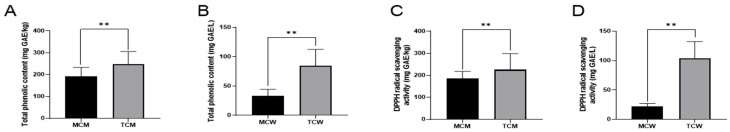
The total phenolic content (**A**,**B**) and DPPH radical scavenging activities (**C**,**D**) of coconut milk and water. “**” indicates the difference is statistically significant (*p* < 0.01).

Phenolic compounds are widely considered as key contributors to the functional properties of coconut, and their concentration can serve as an important indicator of nutritional and bioactive potential. In this study, TPC of all samples was determined using the Folin–Ciocalteu method. As shown in Figure 1A, distinct differences were observed between tender and mature coconuts. In coconut meat, TCM exhibited a significantly higher TPC (248.19 ± 35.89 mg GAE/kg) compared with MCM (190.90 ± 6.94 mg GAE/kg). A similar pattern was evident in coconut water, where TCW contained 92.41 ± 14.83 mg GAE/L and MCW contained 33.26 ± 6.99 mg GAE/L. Statistical analysis revealed that the total phenolic content of mature coconuts, both milk and water, was significantly lower than that of tender coconuts (*p* < 0.01). Specifically, the TPC of TCM was approximately 1.3 times higher than that of MCM, while the TPC of TCW was about 2.7 times higher than that of MCW. These results clearly highlight the significant influence of maturity on the phenolic composition of coconut products. Previous studies support these findings. Li et al. [19] demonstrated that coconut maturity strongly affects its internal constituents, noting that coconuts harvested at around 8 months after flowering contain abundant water and higher sugar levels, making them more suitable for fresh consumption or beverage processing. By contrast, at the mature stage (12 months), coconuts accumulate more fat, protein, and carbohydrates, with a marked thickening of the endosperm and a simultaneous decline in both water content and soluble sugars. This developmental transition is accompanied by a reduction in phenolic compounds, which may be due to the partial utilization of phenolic metabolites in physiological processes such as cell wall fortification. From a functional food perspective, these results suggest that incorporating tender coconut meat or water into the coconut milk production could enhance the phenolic content and bioactivity of coconut-based products.

Given the increasing consumer interest in natural functional foods, the antioxidant capacity of coconut is of particular relevance. The DPPH radical scavenging assay, a widely used method for assessing antioxidant activity, was employed to evaluate the samples (Figure 1B). The results were consistent with TPC values. DPPH scavenging activity was 184.14 ± 7.47 mg GAE/kg in MCM and 225.00 ± 33.25 mg GAE/kg in TCM. For coconut water, the activity was markedly lower, with MCW containing 22.23 ± 2.12 mg GAE/L compared to 106.96 ± 0.80 mg GAE/L in TCW. Again, tender coconuts exhibited significantly stronger antioxidant activity than mature ones (*p* < 0.01), a difference that can largely be attributed to their higher phenolic content. Phenolic compounds exhibit their antioxidant activity through several mechanisms, such as scavenging radicals, chelating metal ions, and inhibiting pro-oxidant enzymes [20]. Therefore, the observed variations in phenolic content between tender and mature coconuts directly translated into differences in antioxidant potential.

Collectively, these findings confirm that maturity exerts a decisive influence on both phenolic levels and antioxidant activity in coconuts. From an application standpoint, supplementation with tender coconut water during product development may provide an effective strategy to enhance the antioxidant functionality of coconut milk–based foods and beverages.

### 3.3. Phytochemical Profile Analysis

**Figure 2:**
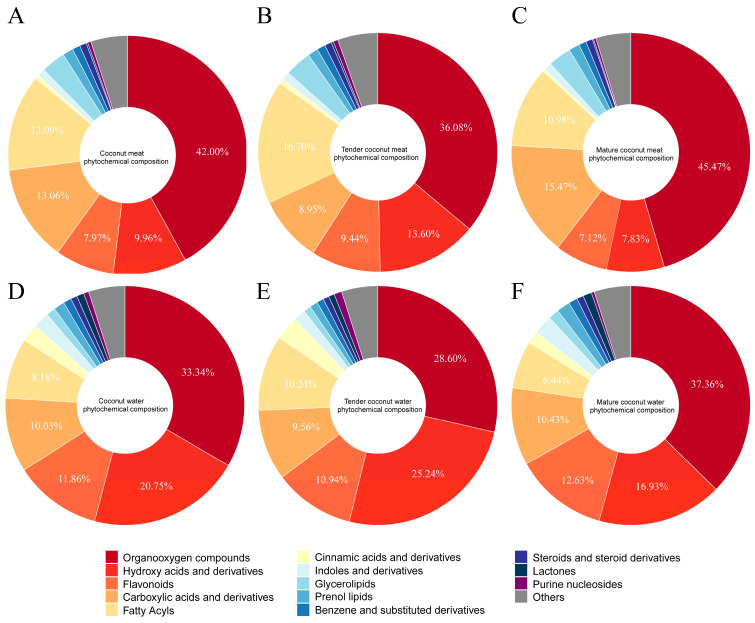
Phytochemical profiles of coconut meat (**A**), tender coconut meat (**B**), mature coconut meat (**C**), coconut water (**D**), tender coconut water (**E**), and mature coconut water (**F**).

The phytochemical composition of coconut milk contributes substantially to its nutritional and functional properties. To gain a comprehensive understanding of these constituents, an untargeted UHPLC-Q-Orbitrap-MS analysis was performed on coconut meat at two developmental stages and their corresponding coconut water samples. The representative chromatograms of the four types of samples are demonstrated in the Supplementary Material Figure S2. Component identification was achieved based on accurate parent ion and fragment ion *m*/*z* information under both positive and negative ionization modes. In total, 1065 phytochemicals were tentatively identified and classified into 13 major categories, as demonstrated in the pie charts of Figure 2. In coconut meat (Figure 2A), the predominant categories (>1% relative abundance) included organooxygen compounds (42.00%, 199 compounds), fatty acyls (13.09%, 130 compounds), carboxylic acids and derivatives (13.06%, 231 compounds), hydroxy acids and derivatives (9.96%, 12 compounds), flavonoids (7.97%, 58 compounds), followed by glycerolipids (3.30%), prenol lipids (1.45%), unsaturated hydrocarbons (1.12%), and benzene derivatives (1.06%). Collectively, the top five categories accounted for approximately 86% of the phytochemical profile. Notably, phenolic compounds (flavonoids, hydroxycinnamic acids and derivatives, and benzoic acids and derivatives) represented 8.58% of total constituents, underscoring their potential nutritional relevance. The phytochemical profile of coconut water exhibited a similar compositional framework, although the relative abundance of key categories differed (Figure 2D). The five most abundant groups were organooxygen compounds (33.34%), hydroxy acids and derivatives (20.75%), flavonoids (11.86%), carboxylic acids and derivatives (10.03%), and fatty acyls (8.18%). Importantly, the overall proportion of phenolic compounds in coconut water reached 14.44%, exceeding that of coconut meat. This suggests that while coconut meat is richer in lipid-related phytochemicals, coconut water has significantly higher content of phenolics.

A comparison between tender and mature coconut meat samples further revealed maturity-dependent compositional differences (Figure 2B,C). In TCM, the top contributors were organooxygen compounds (36.08%), fatty acyls (16.70%), hydroxy acids and derivatives (13.60%), flavonoids (9.44%), and carboxylic acids and derivatives (8.95%). By contrast, MCM was dominated by organooxygen compounds (45.47%), followed by carboxylic acids and derivatives (15.47%), fatty acyls (10.98%), hydroxy acids and derivatives (7.83%), and flavonoids (7.12%). Similar changes were observed in coconut water (Figure 2E,F). These shifts suggest that as coconuts mature, organooxygen compounds and carboxylic acids accumulate, while fatty acyls, hydroxy acids, and flavonoids decline proportionally.

Overall, these findings demonstrate that both the tissue type (meat vs. water) and developmental stage (tender vs. mature) exert significant influences on the phytochemical profile of coconut. Coconut meat is characterized by a higher abundance of lipid-derived metabolites, whereas coconut water contains a greater share of phenolic constituents. Moreover, tender coconut meat retains relatively higher proportions of fatty acyls and flavonoids compared with mature meat, highlighting the impact of maturation on nutritional quality. These compositional insights provide a foundation for evaluating the functional value of coconut-derived products and optimizing their use in plant-based nutrition and functional food development. Notably, 96 phenolic compounds were tentatively identified, accounting for ~7.16% of the total detected phytochemicals. These widespread plant compounds are known to confer numerous health benefits, including antioxidant, anti-obesity, anti-inflammatory, and antibacterial activities. Their presence in both coconut meat and water is therefore of particular nutritional and functional significance. The next section focuses specifically on these phenolic constituents to further elucidate their potential health-promoting properties in coconut-derived products.

### 3.4. Phenolic Profile Analysis

Given the relatively high proportion and biological relevance of phenolic compounds in coconut, this section focuses specifically on their detailed characterization in both coconut meat and coconut water. A total of 96 phenolic compounds were tentatively identified, including 58 flavonoids, 25 hydroxycinnamic acids and derivatives, and 13 benzoic acids and derivatives. Detailed information on these phenolic compounds is shown in Supplementary Table S2. Comparative analyses were subsequently performed to investigate differences between coconut meat and coconut water, as well as between tender and mature stages.

#### 3.4.1. General Comparison of the Phenolic Profile

Flavonoids represented the most abundant phenolic class across all samples. The major flavonoids identified included gardenin B, epicatechin, catechin, procyanidin B5, naringin, and procyanidin B2. Overall, flavonoids accounted for 10.84% of total metabolites, with relative contributions of 7.97% in coconut meat and 11.86% in coconut water. A maturity-dependent decline was observed: flavonoids comprised 9.44% of TCM but only 7.12% of MCM. This decreasing pattern suggests that flavonoid accumulation is more prominent in earlier developmental stages, consistent with their potential role in stress defense and growth regulation.

Hydroxycinnamic acids and derivatives were the second major group of phenolics, with key representatives including 2-hydroxycinnamic acid, 3-caffeoyl-1,5-quinolactone, trans-p-feruloyl-β-D-glucopyranoside, N1,N10-diferuloylspermidine, and caffeoylferuloylspermidine. This class accounted for 1.87% of total metabolites, with a markedly higher abundance in coconut water (2.36%) compared to coconut meat (0.49%). Within meat samples, their proportion declined from 0.66% in TCM to 0.39% in MCM, again indicating a maturity-related reduction. Given their well-established antioxidant and antimicrobial properties, the relatively higher concentration of hydroxycinnamic derivatives in tender coconuts suggests enhanced functional potency at early developmental stages.

Benzoic acids and derivatives were less abundant, with major compounds including benzocaine, ethyl benzoate, bis(2-ethylhexyl) phthalate, (+)-zeylenol, and vanillic acid. They accounted for only 0.19% of total metabolites, with relative levels of 0.22% in coconut water and 0.12% in coconut meat. Within meat samples, benzoic derivatives were slightly higher in TCM (0.13%) than in MCM (0.11%). Despite their low abundance, certain members, such as vanillic acid, are bioactive antioxidants and anti-inflammatory agents, likely contributing to the overall health-promoting attributes of coconut products.

Overall, the phenolic profile revealed clear influences of both tissue type and developmental stage. Coconut water contained a higher proportion of phenolics than coconut meat, particularly flavonoids and hydroxycinnamic acids, highlighting its role as a polyphenol-rich beverage. In addition, tender samples consistently exhibited greater phenolic abundance than mature ones, indicating that phenolic richness declines with fruit maturation as metabolic resources shift toward lipid accumulation in the kernel. Importantly, the identified flavonoids such as catechin, epicatechin, and procyanidins, which are well known for their antioxidant and cardioprotective properties, underscore the nutritional and functional potential of coconut-derived products, especially in their tender stage.

**Figure 3:**
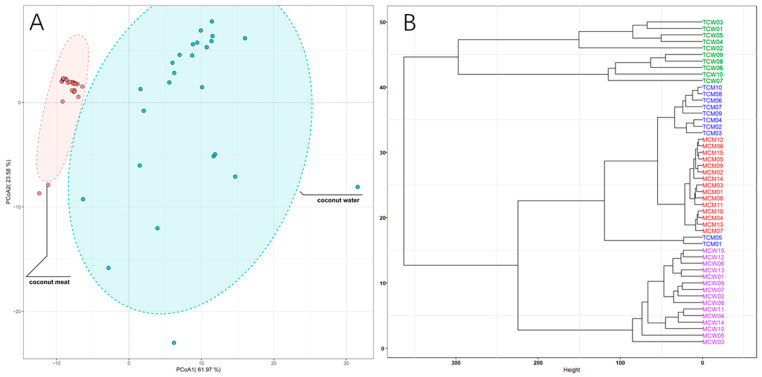
PCoA score plots (**A**) and HCA dendrograms (**B**) of the coconut meat and water samples.

To further elucidate the overall distribution patterns of phenolic compounds across different sample types, principal coordinate analysis (PCoA) and hierarchical clustering analysis (HCA) were conducted (Figure 3). The PCoA score plot (Figure 3A) clearly separated coconut meat and coconut water, indicating a pronounced compositional divergence between these two matrices. Coconut meat samples clustered tightly within a small region, reflecting their relatively homogeneous phenolic composition. In contrast, coconut water samples were more widely dispersed, suggesting greater compositional variability, potentially due to environmental or physiological differences during fruit development. The HCA dendrogram (Figure 3B) further supported these distinctions, revealing different clades corresponding to coconut water and coconut meat. Within the coconut meat group, tender (TCM) and mature (MCM) samples formed two adjacent but distinguishable subclusters, indicating maturity-dependent compositional shifts. For coconut water, tender (TCW) and mature (MCW) samples were also grouped separately, though with partially overlapping branches, reflecting both shared and distinct features in their phenolic profiles. Therefore, PCoA and HCA analyses reinforce that phenolic composition in coconut is strongly influenced by both tissue type and developmental stage. The tight clustering of coconut meat samples indicates greater biochemical stability in kernel-derived phenolics, whereas the wider variation in coconut water points to dynamic metabolite fluctuations during fruit maturation. These findings highlight the complementary contributions of meat and water to the overall phenolic profile of coconut, with coconut water serving as a more variable but phenolic-rich fraction, and coconut meat providing a more stable but lipid-associated phenolic pool.

#### 3.4.2. Differential Phenolics Between Coconut Meat and Coconut Water

**Figure 4:**
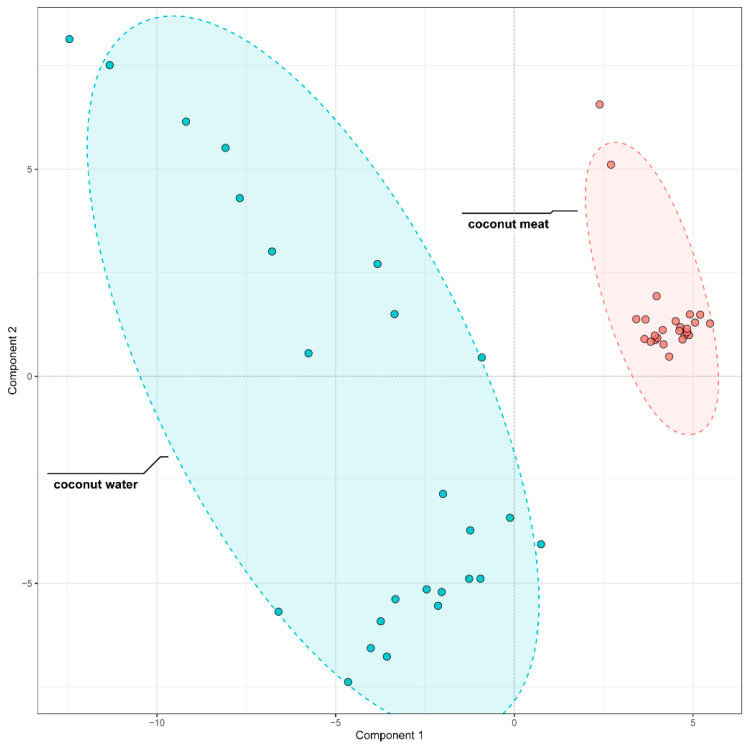
PLS-DA Comparison of phenolic compounds in coconut meat and coconut water.

To further discriminate the phenolic composition of coconut meat and coconut water, we employed partial least squares-discriminant analysis (PLS-DA) on the 96 identified phenolic compounds. The model demonstrated excellent performance, with both its fitting (R^2^) and predictive (Q^2^) values reaching 0.99 during cross-validation, confirming its high reliability and predictive ability. The score plot revealed a distinct separation between the two sample types (Figure 4), confirming that coconut meat and coconut water exhibit markedly different phenolic profiles. The coconut meat samples clustered tightly together, indicating a relatively homogeneous phenolic composition, whereas the coconut water samples exhibited broader dispersion, reflecting greater variability in their phenolic profiles.

**Figure 5:**
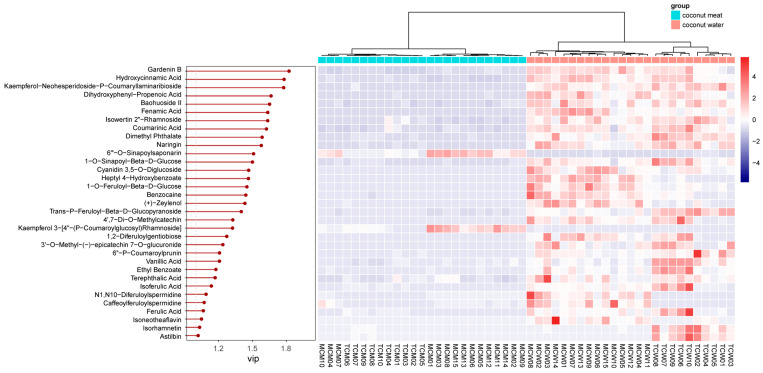
VIP values and relative content of differential phenolics between coconut meat and coconut water.

To identify the specific compounds driving this separation, variable importance in projection (VIP) scores were calculated. A total of 33 compounds showed VIP values greater than 1, indicating their strong contribution to group discrimination. These compounds were further visualized through a heatmap analysis (Figure 5), which highlighted contrasting abundance patterns between coconut meat and coconut water.

Among these, the top five discriminant phenolics were gardenin B (VIP = 1.82), hydroxycinnamic acid (VIP = 1.78), kaempferol-neohesperidoside-p-coumaryllaminaribioside (VIP = 1.78), dihydroxyphenyl-propenoic acid (VIP = 1.66), and baohuoside II (VIP = 1.65). Gardenin B, a polymethoxyflavone, was markedly enriched in coconut water, suggesting a potential role in antioxidant defense and plant–environment interactions [21]. Hydroxycinnamic acid and its derivatives, also more abundant in coconut water, are well known for their antioxidant, antimicrobial, and anti-inflammatory properties [22], further emphasizing the functional potential of water compared with meat. Similarly, kaempferol glycosides and baohuoside II (a prenylated flavonoid) were more prominent in coconut water, indicating that glycosylated and specialized flavonoids preferentially accumulate in the liquid endosperm.

#### 3.4.3. Differential Phenolics Between Tender and Mature Coconut Meat

**Figure 6:**
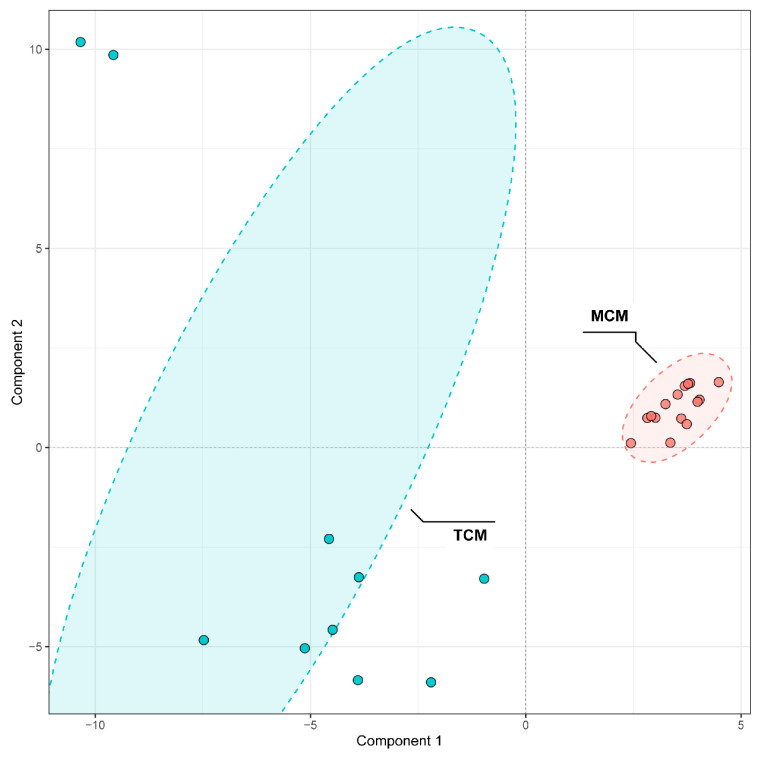
PLS-DA Comparison of phenolic compounds in mature (MCM) and tender (TCM) coconut meat.

To investigate the influence of developmental stage on the phenolic composition of coconut meat, PLS-DA was conducted to differentiate TCM and MCM. The PLS-DA score plot showed a clear separation between TCM and MCM (Figure 6), indicating distinct phenolic signatures associated with the developmental stage. Notably, MCM samples clustered more tightly, indicating a relatively uniform phenolic composition at the mature stage, whereas TCM samples displayed broader dispersion, reflecting greater variability in phenolic accumulation during the earlier developmental stage. This suggests that phenolic biosynthesis and metabolism are more dynamic in tender coconuts but stabilize as the fruit matures.

**Figure 7:**
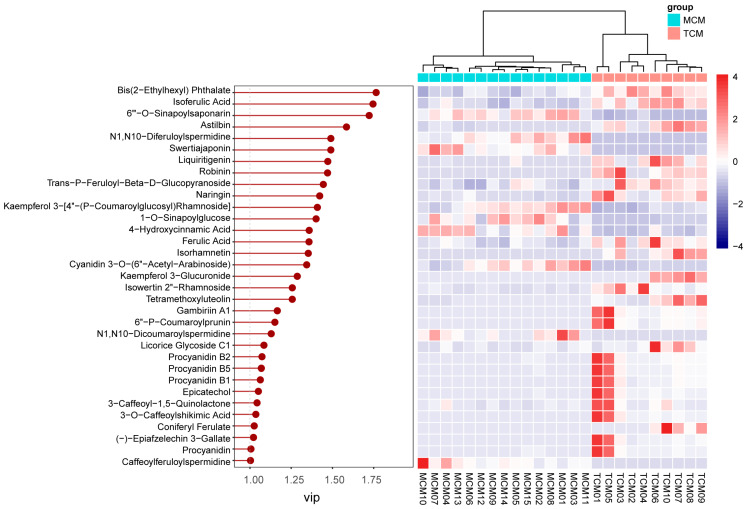
VIP values and relative content of differential phenolics between mature (MCM) and tender (TCM) coconut meat.

VIP analysis identified 33 discriminant compounds with VIP values greater than 1, highlighting their significant contribution to the observed separation. These discriminant phenolics were further visualized in a heatmap (Figure 7), which clearly illustrated maturity-dependent accumulation patterns.

The top five discriminant compounds were bis(2-ethylhexyl) phthalate (VIP = 1.77), isoferulic acid (VIP = 1.75), 6′′′-O-sinapoylsaponarin (VIP = 1.72), astilbin (VIP = 1.58), and N1,N10-diferuloylspermidine (VIP = 1.49). Among these, isoferulic acid, a hydroxycinnamic acid derivative, was more abundant in TCM, indicating stronger accumulation of antioxidant and antimicrobial phenolics in the early developmental stage [23,24]. Similarly, astilbin, a flavonoid with notable anti-inflammatory and hepatoprotective properties [25], was enriched in TCM, further reinforcing its higher functional potential. In contrast, 6′′′-O-sinapoylsaponarin, a phenolic glycoside, and N1,N10-diferuloylspermidine, a hydroxycinnamic acid derivative, were more enriched in MCM, suggesting compositional shifts toward more complex or storage-related phenolics with fruit maturation.

#### 3.4.4. Differential Phenolics Between Tender and Mature Coconut Water

**Figure 8:**
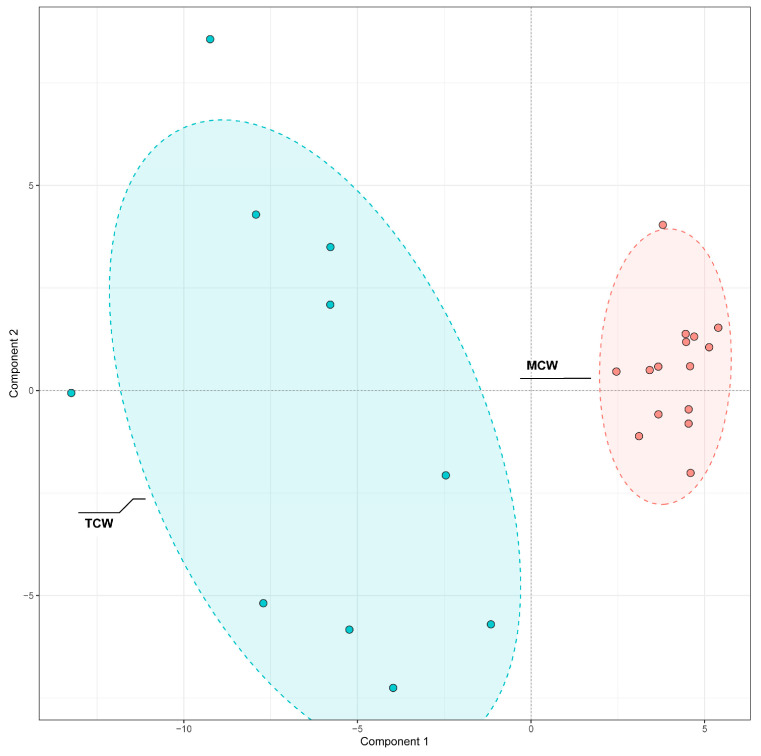
PLS-DA Comparison of phenolic compounds in mature (MCW) and tender (TCW) coconut water.

A similar PLS-DA approach was used to examine differences between TCW and MCW (Figure 8). The clear separation along the first principal component indicated maturity-dependent variation in phenolic composition.

**Figure 9:**
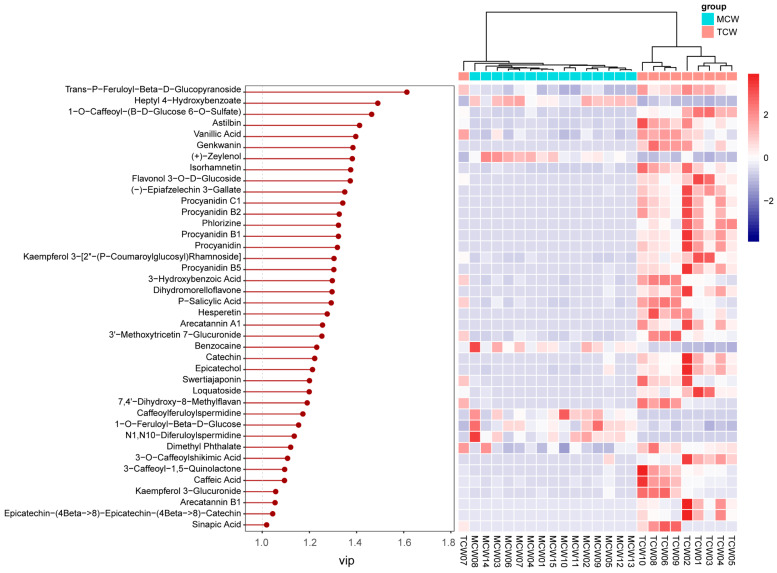
VIP values and relative content of differential phenolics between mature (MCW) and tender (TCW) coconut water.

VIP analysis identified 40 discriminant compounds (VIP > 1), whose abundance patterns were displayed in the heatmap (Figure 9). The differential abundance patterns revealed that several procyanidins (B1, B2, B5, C1) and hydroxybenzoic acid derivatives were more enriched in MCW, whereas TCW was characterized by higher levels of catechins, caffeoyl derivatives, and certain flavonoids such as hesperetin and loquatoside. The five top VIP compounds were trans-p-feruloyl-β-D-glucopyranoside (VIP = 1.61), heptyl 4-hydroxybenzoate (VIP = 1.49), 1-O-caffeoyl-(β-D-glucose 6-O-sulfate) (VIP = 1.46), astilbin (VIP = 1.41), and vanillic acid (VIP = 1.40). These compounds collectively contributed to the clear separation observed between TCW and MCW. Their distribution patterns suggest that the maturation of coconut water is associated with an accumulation of benzoic and cinnamic acid derivatives, possibly reflecting enhanced phenolic metabolism and oxidative polymerization during fruit development.

Overall, the phenolic composition of coconut exhibits remarkable tissue- and maturity-dependent diversity. Coconut water is a richer and more dynamic reservoir of phenolics, particularly flavonoids and hydroxycinnamic acids, while coconut meat has a more stable but lipid-associated matrix. The tender stage of development is characterized by higher concentrations of bioactive phenolics such as catechins, epicatechin, and astilbin, conferring stronger antioxidant potential, whereas the mature stage features a transition toward structurally complex or storage-related phenolics. These compositional transitions reflect the metabolic reprogramming accompanying coconut maturation and underline the nutritional and functional significance of phenolic compounds, particularly in tender coconut products.

## 4. Conclusions

This study provides a comprehensive analysis of the nutritional and functional composition of the raw materials of coconut milk, including coconut meat and water, highlighting their potential as plant-based milk alternatives. Fatty acid profiling revealed that mature coconut meat, the primary raw material for coconut milk, is enriched in medium-chain saturated fatty acids, supporting its creamy texture and energy-dense profile. Tender coconut water, in contrast, exhibited significantly higher phenolic content and antioxidant activity, offering additional functional benefits. Metabolomic analyses further confirmed tissue- and maturity-dependent variations in phytochemicals, particularly phenolics such as catechin, epicatechin, and astilbin. These findings indicate that integrating tender coconut water or selecting appropriate maturity stages can enhance the nutritional and bioactive properties of coconut milk. Overall, coconut milk emerges as a versatile plant-based alternative, combining lipid-rich nutrition from mature meat with potential antioxidant enhancement from tender water, providing valuable guidance for functional food development and optimization of plant-based dairy substitutes.

## Figures and Tables

**Table 1:** Fatty acid profile of coconut meat and water samples.

	Fatty Acid Content				Fatty Acid Percentage			
	Coconut Meat		Coconut Water		Coconut Meat		Coconut Water	
	MCM (g/kg)	TCM (g/kg)	MCW (g/100 mL)	TCW (g/100 mL)	MCM (%)	TCM (%)	MCW (%)	TCW (%)
Caproic acid (C6:0)	1.11 ± 0.24 *	0.32 ± 0.05	nd	nd	0.37	0.35	-	-
Caprylic acid (C8:0)	16.81 ± 1.59 *	2.98 ± 0.81	0.10 ± 0.10	nd	5.61	3.28	2.4	-
Capric acid (C10:0)	18.94 ± 1.46 *	3.24 ± 0.90	0.18 ± 0.04 *	0.02 ± 0.02	6.32	3.57	4.87	1.81
Lauric acid (C12:0)	142.97 ± 4.99 *	35.11 ± 5.49	1.50 ± 0.05 *	0.22 ± 0.03	47.7	38.64	41.88	24.64
Myristic acid (C14:0)	57.39 ± 2.08 *	18.17 ± 0.61	0.67 ± 0.03 *	0.33 ± 0.07	19.15	20	18.56	18.65
Palmitic acid (C16:0)	29.79 ± 1.89 *	12.53 ± 2.22	0.70 ± 0.01 *	0.05 ± 0.03	9.94	13.79	19.4	27.38
Oleic acid (C18:1)	3.24 ± 0.38	4.30 ± 2.35	0.12 ± 0.03	0.12 ± 0.02	1.08	4.72	3.34	4.21
Linoleic acid (C18:2)	16.09 ± 1.97 *	11.24 ± 3.25	0.26 ± 0.01 *	0.16 ± 0.05	5.37	12.37	7.11	9.6
Stearic acid (C18:0)	13.51 ± 2.40 *	3.18 ± 1.27	0.11 ± 0.07 *	0.60 ± 0.13	4.51	3.5	3.06	13.71
MCFAs	178.35 ± 5.49 *	41.34 ± 7.95	1.80 ± 0.41 *	0.17 ± 0.04	59.51	45.49	50.14	49.82
UFAs	18.40 ± 2.16 *	15.53 ± 5.60	0.38 ± 0.03 *	0.21 ± 0.15	6.14	17.09	10.45	13.81
SFAs	281.40 ± 2.25 *	75.34 ± 5.61	3.20 ± 0.22 *	1.00 ± 0.04	93.89	82.91	90.13	86.19
Total	299.70 ± 16.98 *	90.87 ± 16.86	3.60 ± 0.20 *	1.20 ± 0.20	100	100	100	100

nd: not detected; values labeled with “*” indicate significantly different from the corresponding tender materials (*p* < 0.05).

## Data Availability

The original contributions presented in the study are included in the article/Supplementary Material, further inquiries can be directed to the corresponding author.

## Supplementary Materials

The following supporting information can be downloaded at: https://www.mdpi.com/article/10.3390/foods14234023/s1, Figure S1. GC-MS chromatograms of the four types of coconut milk raw materials, including mature coconut meat (MCM), tender coconut meat (TCM), mature coconut water (MCW), and tender coconut water (TCW). Figure S2. UHPLC-Q-Orbitrap-MS chromatograms of the four types of coconut milk raw materials, including mature coconut meat (MCM), tender coconut meat (TCM), mature coconut water (MCW), and tender coconut water (TCW), in negative ion mode (A) and positive ion mode (B). Table S1. Calibration curves of fatty acids. Table S2. Detailed information of the identified phenolic compounds.

## Abbreviations

The following abbreviations are used in this manuscript:

MCM	Mature coconut meat
TCM	Tender coconut meat
TCW	Tender coconut water
TPC	Total phenolic content
DPPH	1,1-diphenyl-2-trinitrohydrazine
UHPLC-Q-Orbitrap-MS	Ultrahigh-performance liquid chromatography coupled with quadrupole-Orbitrap high-resolution mass spectrometry
CAGR	Compound annual growth rate
MCFA	Medium-chain fatty acid
LCFA	Long-chain fatty acid
LC–MS	Liquid Chromatography–Mass Spectrometry
GC–MS	Gas chromatography–mass spectrometry
MCW	Mature coconut water
GAE	Gallic acid equivalents
LC-HRMS	Liquid Chromatography–High Resolution Mass Spectrometry
ESI	Electrospray ionization
SD	Standard deviation
PCoA	Principal coordinate analysis
HCA	Hierarchical cluster analysis
PLS-DA	Partial least squares-discriminant analysis
SFA	Saturated fatty acid
UFA	Unsaturated fatty acid
VIP	Variable importance in projection
