# Supplementary material for: Comparative Study of the Fatty Acid and Phenolic Profiles of Tender and Mature Coconut for Coconut Milk Production
Source: Foods. 2025 Nov 24;14(23):4023. doi: 10.3390/foods14234023 (PMC12691760; doi:10.3390/foods14234023)
Supplement: Supplementary file 1 [file foods-14-04023-s001.zip › Supporting information.pdf]

Comparative Study of the Fatty Acid and Phenolic Profiles of Tender and Mature Coconut for Coconut Milk Production

Supporting information

Rongqian Jiang, Danpeng Xue, Yanqing Chen, Xucong Lv, Li Ni, Zhibin Liu *

Institute of Food Science & Technology, Fuzhou University, Fuzhou, Fujian, 350108, China

*Corresponding author:

Dr. Zhibin Liu, Institute of Food Science & Technology, Fuzhou University, No. 2 Xueyuan Road, Fuzhou 350108, China

Email: liuzhibin@fzu.edu.cn

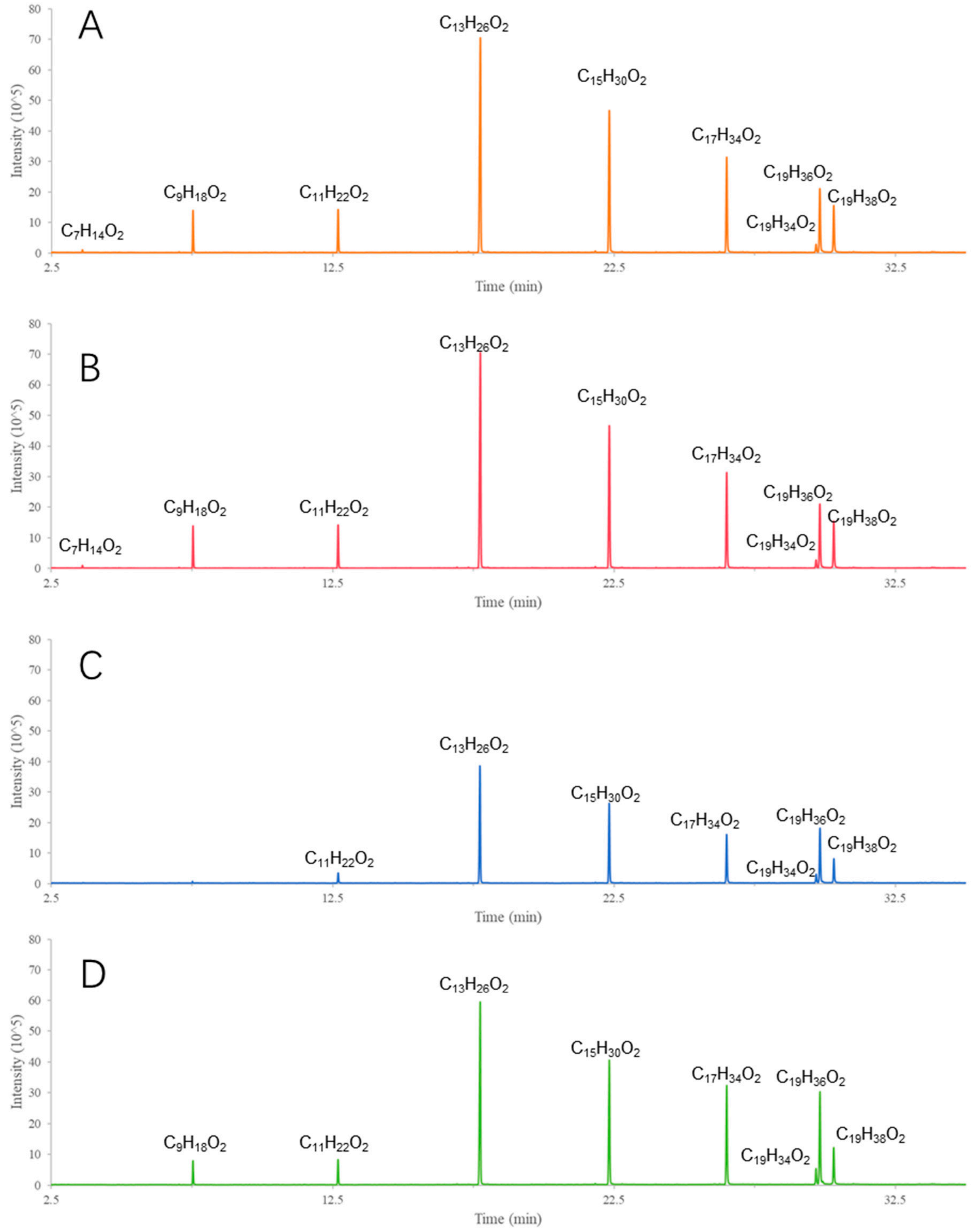

Figure S1. GC-MS chromatograms of the four types of coconut milk raw materials, including mature coconut meat (MCM), tender coconut meat (TCM), mature coconut water (MCW), and tender coconut water (TCW).

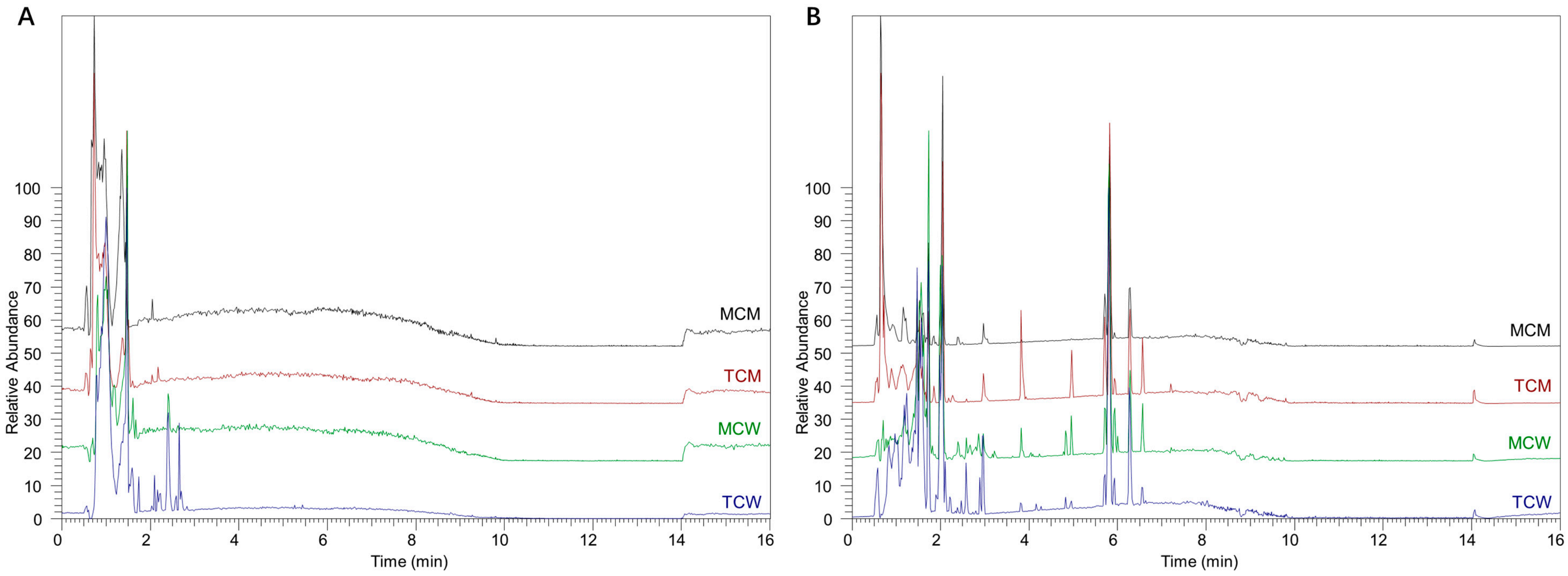

Figure S2. UHPLC-Q-Orbitrap-MS chromatograms of the four types of coconut milk raw materials, including mature coconut meat (MCM), tender coconut meat (TCM), mature coconut water (MCW), and tender coconut water (TCW), in negative ion mode (A) and positive ion mode (B).

Table S1. Calibration curves of fatty acids.

Fatty acid methyl esters	Retention time	Calibration curve	R^2
Hexanoic acid, methyl ester	3.616	Y=0.000403*X + 19.44	0.991
Octanoic acid, methyl ester	7.550	Y=0.000361*X + 26.92	0.993
Decanoic acid, methyl ester	12.707	Y=0.000337*X + 36.32	0.983
Dodecanoic acid, methyl ester	17.754	Y=0.000298*X + 57.75	0.994
Methyl myristoleate	22.343	Y=0.000272*X + 24.21	0.994
Hexadecanoic acid, methyl ester	26.521	Y=0.000225*X + 76.65	0.995
9,12-Octadecadienoic acid (Z,Z)-, methyl ester	29.702	Y=0.000269*X + 39.98	0.990
9-Octadecenoic acid, methyl ester, (E)-	29.836	Y=0.000185*X + 39.56	0.960
Methyl stearate	30.331	Y=0.000175*X + 86.16	0.993
